
JOURNAL INFORMATION
==============================
NLM Title Abbreviation: ScientificWorldJournal
Journal ID: ScientificWorldJournal
NLM Journal ID: 101131163
Journal ID: TSWJ

The Scientific World Journal

ISSN: 2356-6140
EISSN: 1537-744X
Publisher: Wiley

ARTICLE INFORMATION
==============================
PMCID: PMC6084183
PMID: 12805675
DOI: 10.1100/tsw.2001.38
Article ID: 989352
Article version: 1
Subjects: Directions in Science

Finding the Stuff that Dreams are Made Of

Stickgold Robert
Harvard Medical School, Boston, MA 02115, USA
Publication date: 2001
Electronic publication date: 2001 May 11
Volume: 1
First page: 211
Last page: 212
Copyright: Copyright © 2001 Robert Stickgold.
Copyright year: 2001
License: This is an open access article distributed under the Creative Commons Attribution License, which permits unrestricted use, distribution, and reproduction in any medium, provided the original work is properly cited.
License URL: https://creativecommons.org/licenses/by/3.0/

Keywords: sleep, REM sleep, memory

==============================
Directions in Science
*TheScientificWorld* (2001) 1, 211–212
ISSN 1532-2246; DOI 10.1100/tsw.2001.38

### Finding the Stuff that Dreams are Made Of

Robert Stickgold
*Harvard Medical School, 74 Fenwood Rd., Boston MA 02115*

**KEY WORDS:** sleep, REM sleep, memory

**DOMAINS:** neuroscience, learning and memory, behavior

The scientific study of dreams has had a long but tortured history. While the discovery of REM sleep in 1953 [1] and its strong correlation with dreaming [2] led to a renewed hope that the study of dreaming could be moved to a solidly scientific and physiological base, such studies have provided only mixed success. In 1977, Hobson and McCarley [3] proposed the activation-synthesis model for dream construction based on the physiological features of REM sleep, but since then the field has shown surprisingly little progress.

Part of the reason for this slow progress has been the absence of a powerful experimental paradigm for studying dream content; there has been no way to reproducibly elicit a specific dream or dream feature with a specific experimental manipulation. Or at least not until now. In a recent paper in the journal *Science*, Stickgold and his colleagues [4] used daytime playing of the computer game Tetris to induce sleep onset dreams containing images from the game. Seventy-five percent of novices and fifty percent of experienced players reported such “hypnagogic dreams” during forced awakenings performed over two or three nights following days on which the subjects played Tetris. Most striking, however, was the finding that densely amnesiac patients with bilateral damage to medial temporal lobe structures including the hippocampus, while unable to recall playing Tetris from one session to the next (or to remember the experimenter or why the computers were in their bedrooms), still produced hypnagogic dreams of Tetris. Indeed, the content of these dreams was identical to that of the normals as were both the prevalence (60% of patients, 62% of normals) and frequency (7.4% of all reports from patients; 7.2% from normals) of these reports. These findings suggest that the hippocampal, episodic memory system does not normally contribute to the construction of hypnagogic dreams.

The study of hypnagogic dreams has fallen on hard times since the pioneering work of Foulkes and Vogel in 1965 [5], although a recent paper by Rowley and his colleagues [6] indicated a renewed interest in the field. Now, by showing that experimental interventions can reliably shape the content of hypnagogic dreams, we might see a shift of some of the attention normally focused on REM sleep dreaming to this intriguing early night sleep phase. The fact that hippocampal involvement appears to be minimal or even completely absent in the construction of these dreams may be relevant to REM sleep dreaming as well, since studies in animals have suggested that the flow of information from the hippocampus to the neocortex is shut off during REM sleep [7]. This similarity raises the possibility that studies of hypnagogic dreaming may be a valuable source of information about REM sleep dreaming as well.

Several specific findings in the *Science* article provide food for thought. First, the actual images reported were very consistent across subjects and across groups. All subjects reported seeing Tetris pieces floating down in front of their eyes, sometime being rotated and occasionally fitting in with other pieces. (Amnesiacs referred to “those shapes”, or “little squares coming on a screen” or “images turned on their sides…like blocks.”) But no one reported seeing the background picture or scoreboard from the computer screen, or the computer or keyboard, or the desk or room in which they played. Thus, the images were not episodic memories, but extracted and abstracted elements from the memories. Second, 90% of the hypnagogic images of Tetris reported by novices occurred on the second night of recording, with only 10% on the first night. This suggests that the brain is using a complex algorithm in identifying waking images to be replayed at sleep onset. Third, two of the five experienced players who reported Tetris images reported that they

E-mail: Robert_stickgold@hms.harvard.edu
© 2001 with author.

were not from the version of Tetris used in the current study, but from the version they had played in their youth. Thus, the images were not simply recent perceptual traces, but included associated memories, in one case from at least five years earlier. Finally, and most tantalizing, in each of the three groups, the subjects who performed most poorly during the initial 2-hr training and practice session were most likely to report Tetris images. While the significance of these correlations is uncertain, they add to a growing literature suggesting a role for sleep in memory consolidation and integration [8-11].

The major implications of this study are twofold. First, by demonstrating that normal hypnagogic dreams are produced in the presence of massive medial temporal lobe damage, Stickgold and colleagues have demonstrated that hippocampal processes are not normally involved in the construction of these dreams. Since images from years earlier can be evoked, it appears that the construction of these dreams is a nearly pure neocortical process. Second, and perhaps of larger general significance, the ability to reliably produce very similar dreams across subjects through experimental manipulation for the first time permits a scientific investigation of the factors controlling the incorporation of memories into dream material.

### REFERENCES

1. Aserinsky, E. and Kleitman, N. (1953) Regularly occurring periods of ocular motility and concomitant phenomena during sleep. *Science* **118,** 361–375.
2. Dement, W. and Kleitman, N. (1957) The relation of eye movements during sleep to dream activity: an objective method for the study of dreaming. *J. Exp. Psychol.* **53,** 339–346.
3. Hobson, J.A. and McCarley, R.W. (1977) The brain as a dream-state generator: an activation-synthesis hypothesis of the dream process. *Am. J. Psychiatry* **134,** 1335–1348.
4. Stickgold, R. et al. (2000) Replaying the game: hypnagogic images in normals and amnesics. *Science* **290,** 350–353.
5. Foulkes, D. and Vogel, G. (1965) Mental activity at sleep onset. *J. Abnormal Psychol.* **70,** 231–243.
6. Rowley, J.T., Stickgold, R., and Hobson, J.A. (1998) Eyelid movements and mental activity at sleep onset. *Consciousness Cogn.* **7,** 67–84.
7. Buzsáki, G. (1996) The hippocampo-neocortical dialogue. *Cereb. Cortex* **6,** 81–92.
8. Stickgold, R. (1998) Sleep: off-line memory reprocessing. *Trends Cogn. Sci.* **2**(**12**), 484–492.
9. Stickgold, R., James, L., and Hobson, J.A. (2000) Visual discrimination learning requires post-training sleep. *Nature Neurosci.* **2**(**12**), 1237–1238.
10. Stickgold, R. (2001) EMDR: a putative neurobiological mechanism of action. *J. Clin. Psychol*. **57**, in press.
11. Smith, C. (1995) Sleep states and memory processes. *Behav. Brain Res.* **69,** 137–145.

**This article should be referenced as follows:**

Stickgold, R. (2001) Finding the stuff that dreams are made of. *TheScientificWorld* 1, 211–212.

| | | |
|---|---|---|
| Received | February | 20, 2001 |
| Accepted | March | 26, 2001 |
| Published | May | 11, 2001 |
